# A Dual-Channel Acquisition Method Based on Extended Replica Folding Algorithm for Long Pseudo-Noise Code in Inter-Satellite Links

**DOI:** 10.3390/s18061717

**Published:** 2018-05-25

**Authors:** Hongbo Zhao, Yuying Chen, Wenquan Feng, Chen Zhuang

**Affiliations:** Electronic and Information Engineering Department, Beihang University, Beijing 100191, China; amisyy@sina.cn (Y.C.); buaafwq@buaa.edu.cn (W.F.); zhuangchen0214@126.com (C.Z.)

**Keywords:** inter-satellite link, mean acquisition time, dual-channel, folding method, long code

## Abstract

Inter-satellite links are an important component of the new generation of satellite navigation systems, characterized by low signal-to-noise ratio (SNR), complex electromagnetic interference and the short time slot of each satellite, which brings difficulties to the acquisition stage. The inter-satellite link in both Global Positioning System (GPS) and BeiDou Navigation Satellite System (BDS) adopt the long code spread spectrum system. However, long code acquisition is a difficult and time-consuming task due to the long code period. Traditional folding methods such as extended replica folding acquisition search technique (XFAST) and direct average are largely restricted because of code Doppler and additional SNR loss caused by replica folding. The dual folding method (DF-XFAST) and dual-channel method have been proposed to achieve long code acquisition in low SNR and high dynamic situations, respectively, but the former is easily affected by code Doppler and the latter is not fast enough. Considering the environment of inter-satellite links and the problems of existing algorithms, this paper proposes a new long code acquisition algorithm named dual-channel acquisition method based on the extended replica folding algorithm (DC-XFAST). This method employs dual channels for verification. Each channel contains an incoming signal block. Local code samples are folded and zero-padded to the length of the incoming signal block. After a circular FFT operation, the correlation results contain two peaks of the same magnitude and specified relative position. The detection process is eased through finding the two largest values. The verification takes all the full and partial peaks into account. Numerical results reveal that the DC-XFAST method can improve acquisition performance while acquisition speed is guaranteed. The method has a significantly higher acquisition probability than folding methods XFAST and DF-XFAST. Moreover, with the advantage of higher detection probability and lower false alarm probability, it has a lower mean acquisition time than traditional XFAST, DF-XFAST and zero-padding.

## 1. Introduction

Inter-satellite links play an important role in satellite navigation systems, where they provide a direct link within the space segment without the need of an intermediate ground segment to relay the data. Spread spectrum technology is widely applied in present-day inter-satellite link systems. A common form of spread spectrum is direct sequence spread spectrum (DSSS) where incoming binary data is multiplied by a pseudo-noise (PN) code before they are transmitted [1]. Since long PN-codes achieves higher tolerance for jamming and spoofing, and greater multiple access capabilities compare with short codes, ranging mode of inter-satellite link in GPS and BDS systems both adopt the long code spread spectrum system [2,3].

Among the signal synchronization steps of spread spectrum technology, code acquisition is one of the most critical functions of receivers [4]. It is of great significance to study the acquisition of long PN-code. However, with the code period getting longer, the time cost and burdens on hardware are becoming unacceptable [5,6].

The inter-satellite link adopts time-division-multiple-access (TDMA) technology. Each link is assigned a short time-slot, which means signal acquisition and tracking need to be completed in a short time [7]. Moreover, inter-satellite link is characterized by complex electromagnetic interference and low signal-to-noise ratio (SNR). Interference and noise bring great challenges to exact acquisition [8,9]. With the rapid development of the inter-satellite link, an essential concern is how to ensure rapid and exact acquisition.

Long code acquisition methods perform in time or frequency domain depending on how the correlation is done. There are some other methods that estimate the initial states of long PN-code generating shift registers similar to channel decoding algorithms [10,11]. However, these methods converge slowly at low SNR and the applications of them are limited to few types of PN codes like gold codes.

In time domain methods, each correlator can search only few code phases, and using multiple correlators would occupy extra hardware resources. Serial search (SS) [12,13] is one typical method in the time domain which detects code phases one by one. Many tests are required and thus the process is too long. Reference [14] proposes an improved double dwell search scheme that uses a 2D compressed correlator (TDCC) in the 1st dwell search and the convention correlator in the 2nd dwell search. But Signal-to-noise ratio (SNR) deterioration arises as the compression rate is increased.

With great advances in digital processing boards, algorithms based on frequency domain search are more investigated [15]. These methods exploit the property of Fourier transform. Many methods are proposed based on FFT search. Zero-Padding method (ZP) is a popular one among them. The method adds $\frac{N}{2}$ zeros to extend the incoming samples to length *N*. With fast Fourier transform (FFT) technique, ZP can search $\frac{N}{2}+1^{2}$ code phases in parallel. Many methods have been developed based on the ZP scheme [16]. Li Hong [17] proposed a method named Generalized Zero Padding(GZP) that optimizes the length of zeros according to SNR. Ping Jun [18] proposed another modified ZP method that applies interpolation to achieve higher acquisition accuracy. These methods have reasonable performance for Doppler and jamming mitigation but are still inadequate for long codes.

Among proposed long code acquisition methods, folding local PN-code and averaging input and local samples are mostly used in practice. A typical example of the former is XFAST proposed by Yang Chun [19] and the latter is known as Direct Average Method(DAM) [20]. For XFAST, an incoming code segment is simultaneously compared with local code segments to find a match. The use of circular correlation via FFT to determine if a match occurs permits the simultaneous search through all code phase across multiple code segments. Once a match is determined, the location within any reference segment is then determined in a second conventional correlation on a segment to segment basis. The principles of folding methods and averaging methods are similar. By folding local blocks or summing up local samples for average, the code phases to be searched are directly decreased proportional to local folding/averaging number. As more samples are folded or summed up for averaging, more codes take part in a single correlation. Currently, some approaches have been presented specially to modify XFAST or DAM [21,22,23]. Although these methods can reduce acquisition time, Signal-to-noise ratio (SNR) deterioration resulting from folding or summing leads to the decline of detection probability. Moreover, false alarms resulted from peak position ambiguity also lengthen the mean acquisition time.

To address these problems, Feng [24] proposed a dual-channel acquisition approach. This method applies two channels to make correlations. Input samples are divided into blocks with half of local block length and zero-padded to full length prior to FFT. It uses complete and partial peaks for acquisition verification and thus obtains lower false alarm probability. This method works well in low SNR and high dynamic circumstances. However, as the magnitude of partial correlation peak decreases, it becomes more likely to be submerged by noise, affecting acquisition performance. More importantly, this approach takes much more time to synchronize long PN-codes than folding methods like XFAST if code Doppler is not serious enough. Due to the requirement of fast and accurate acquisition in inter-satellite link, unacceptable time cost and unstable detection performance of dual-channel restricted its practical use in inter-satellite link.

Li Hong [25] also proposed a Dual-Folding (DF-XFAST) to improve acquisition performances in inter-satellite link. This method suggests both incoming samples and local samples are folded to obtain a better SNR. Benefiting from the tradeoff between extending coherent integration time and the degraded PN-code correlation properties, DF-XFAST shows its enhancement when code phase difference between input and local codes are integer multiple of overlapping number. However, with equal times of folding and averaging, DF-XFAST method and direct average method are of the same performances, and this assertion is valid only when phase difference between incoming and local signals are just integer multiple of overlapping number. Correlation peak declines linearly as relative phase difference widens, which largely restricted its application.

Based on the above-mentioned methods, many methods have been developed. Wang [26] proposed a method named Auto Switch Approach(AS). By designing a shift module which responds to Doppler and SNR, the method combines DF-XFAST and dual-channel to adapt to tougher situation. The method outperforms both DF-XFAST and dual-channel method, but it requires more hardware resource at the same time. Borna [27] proposed a method named Enhanced Dual Folding(EDF), the method improves DF-XFAST by applying GZP on it. The method is better for high Doppler conditions at the cost of less parallel searching capability than DF-XFAST. Lately, [28] they proposed another acquisition method, which applies DF-XFAST for coarse acquisition and proposes three methods for fine acquisition. The proposed methods for fine acquisition are almost better than ZP in every aspect. However, both EDF and this method have not solved the problem of DF-XFAST.

This paper provides a dual-channel acquisition method based on extended replica folding algorithm (DC-XFAST) to improve detection performance. DC-XFAST method uses a two-channel parallel architecture to make correlation. Each channel contains an incoming signal block. The entire local code segment is folded to have half of the incoming signal block length, and folding local replicas are zero-padded to full length. After circular FFT operation, correlation results contain two peaks of the same magnitude and specified relative position.

The following steps consist of two levels. In the first level, the max-value tactic is adopted and the positions of the two highest peaks are preserved. The second level verifies the result in the first level. The probability that noise values on any certain position has largest magnitude is low, and therefore the false alarm probability is reduced.

Multi-peak verification and max-value detection bring better acquisition performance than the conventional methods. Numerical results show that DC-XFAST has better performance in certain false alarm probabilities compared with zero-padding, XFAST and Dual-Folding method.

The rest of this paper is organized as follows: the second part will be detailed description of DC-XFAST, including system model and observation window of the method. In the third part, the performance is analyzed, including detection probabilities, false alarm probabilities and mean acquisition time, theoretical results are also presented. Numerical results are provided in the fourth part. The discussion of the application is in the fifth part and the conclusion is presented in the sixth.

## 2. Method Description

**Figure 1:**
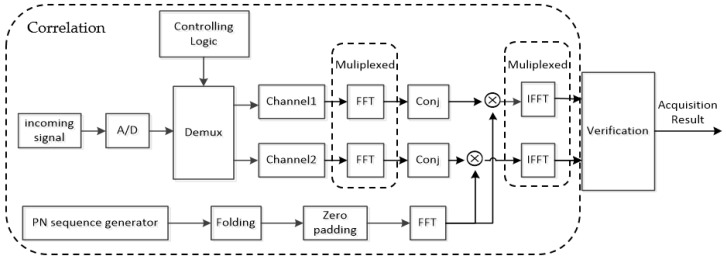
System model of DC-XFAST.

As mentioned in Section 1, the advantage of methods based on folding and averaging is poor since SNR deterioration causes a decline of detection probability and an increased false alarm probability. In addition, the time cost of other unfolding methods such as ZP and dual-channel is unacceptable. For these reasons, the proposed DC-XFAST method folds local samples for fast acquisition, uses multi-peak verification to guarantee low false alarm probability and applies max-value detection to achieve high detection probability. As shown in Figure 1, the steps of the overall algorithm are as follows:

Step 1. The incoming baseband signal is sampled by analog-to-digital converter at an appropriate rate and properly compensated for Doppler offset, then delivered to two channels.

Step 2. Choose FN local code samples, fold them into N new samples, and zero-pad N new samples to 2N.

Step 3. Feed the local blocks into FFT.

Step 4. Group the baseband samples of both channels into blocks of 2N, feed it into FFT, conjugated the FFT results and multiplied with the FFT results of Step 3.

Step 5. Perform IFFT on multiplication result of Step 4, the correlation result of each channels contains two peaks, find the two largest value in the absolute values of IFFT output for each channel. Their positions are preserved for verification.

Step 6. Verify according to the position results in Step 5. If the validation condition is not met, shift (F−4)N+1 unfolded local samples and follow the same process again. When the condition is met, acquisition is over.

In Step 2, we discussed about how local block is constructed. The above steps set forth a two-level acquisition. In the first level, the max-value tactic is adopted and the positions of the two highest peaks are preserved. The second level verifies the result in the first level. In this step, only the relative positions of the peaks are of interest.

**Figure 2:**
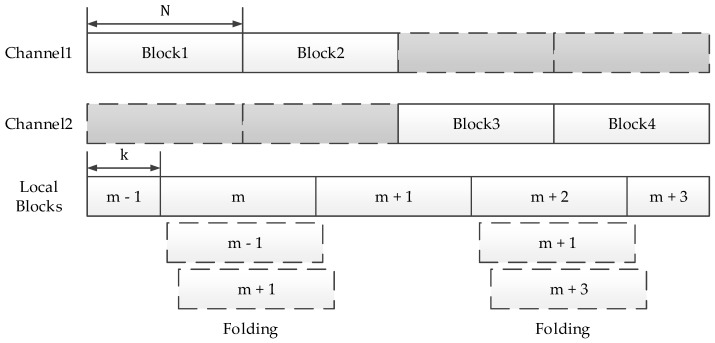
Input blocks and local blocks, each with length N. Local channel 1 is composed of Block 1 and Block 2, channel 2 is 3 and 4. The offset between input block and local block is k, k is uniformly distributed in [1, N]. Block m − 1 to block m + 3 are added together term by term to produce a new block. And the new block is zero padded to 2N.

Step 5 and Step 6 in the above procedure deserve further discussion. In Step 5, we mentioned that the correlation result in each channel contains two peaks. To represent a clearer correlation process of the proposed method, the observation window is shown in Figure 2. The solid lines of channel 1 and channel 2 indicates two incoming signal blocks with length N.

As plotted in Figure 2, the block length N is adjustable in our method. A bigger block would result in a longer coherent length, and higher detection probability. Block m and block m + 2 are fully contained in channel, block m − 1, m + 1, m + 3 are partially contained in channel. Theoretically, ‘fully contained’ blocks will produce full peaks, ‘partially contained’ blocks will produce partial peaks.

In the case of channel 1, the block m has code phase lead k compared with the incoming signal block in channel 1, and block m + 1 has code phase lead (N+k). Therefore, correlation peaks produced by block m and block m + 1 would occur at k and (N+k) respectively. The code phase difference between block m − 1 and the incoming signal block is −k. After circular correlation, the correlation peak produced by block m − 1 would occur at (N+k). The locations of correlation peak produced by block m − 1 and m + 1 are the same.

As we mentioned in Step 2, local blocks are added together term by term to produce a new block with length N, then zero pad the new block to 2N. As a result, the partial peaks of m − 1 and m + 1 are stacked together and produce a full peak, m + 1 and m + 3 each produce another full peak.

As can be deduced from Figure 2, the magnitudes of the partial peaks produced by block m − 1 and block m + 1 are k and N − k respectively. Therefore, the magnitude of the full peak that contains two partial peaks, is the same as the full peak produced by “fully contained” block.

**Figure 3:**
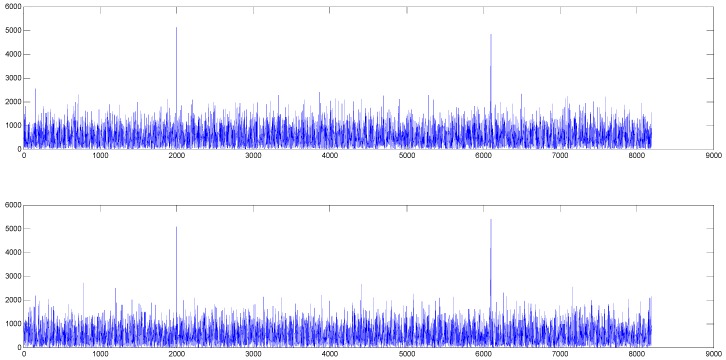
Correlation peaks on condition that SNR equals −10 dB, N = 4096 F = 8 and k = 200.

Correlation results between two channels and local blocks are illustrated in Figure 3, which confirms our theoretical analysis. In Figure 3, x-axis shows sample offsets and y-axis shows correlation peaks. The upper graph is correlation result between channel 1 and the local block, the first peak of which is the full peak produced by “fully contained” block m, and the second peak contains two partial peaks produced by m − 1 and m + 1. Similarly, the lower one is between channel 2 and the local block, the first peak is produced by block m + 1 and the second peak is produced by m + 1 and m + 3. The two peaks in the two correlation results are not strictly consistent due to signal noise.

The verification in Step 6 starts with counting the number of peaks that have the same position or relative position of N. This number is denoted by $N_{s}$ and defined as verification times. As there are four positions of peak preserved in Step 4, the threshold number $N_{\mathrm{th}}$ is confined in set {1, 2, 3, 4}. When $N_{s}\geq N_{\mathrm{th}}$, the acquisition is over. The bigger $N_{\mathrm{th}}$ is chosen, the smaller detection probabilities and false alarm probabilities are obtained. When $N_{\mathrm{th}}=1$ and $N_{\mathrm{th}}=2$ or relative position of two peaks is 0, post-process is needed to determine whether the peak is produced by “fully contained” block or two “partially contained” blocks.

## 3. Theoretical Analysis

This part presents an approximate analysis of the performance of the proposed method DC-XFAST. The formulation considers signal in the presence of thermal noise without multi-access interference. To simplify the analysis, only delays at whole chips are considered in the correlation process. Also, the factor of residual Doppler is neglected. The simplification can be removed without affecting the theoretical analysis below.

Firstly, the correlation method and the distribution of the correlation results are presented. Under simplifying assumptions, detection probabilities of different threshold numbers are derived through joint distribution of the items in correlation results. False alarm probabilities of each threshold numbers are then calculated as a function of block length N and basic false alarm probability based on max-value detection. A common formula of mean acquisition time is also given to analyze the performance of the method.

### 3.1. Correlation Method

The incoming data sample can be written as:

(1) $$s_{l}=\left({\mathrm{Ad}}_{l}c_{l}+n_{l}\right)e^{\left[\left(\frac{2{\pi f}_{d}}{f_{s}}\right)l+\varphi \right]}$$

where A is the signal amplitude, $d_{l}\in \left(-1,\;1\right)$ the modulation data, $c_{l}\in \left(-1,\;1\right)$ PN-code, $f_{d}$ residual frequency and $f_{s}$ sampling frequency, φ random carrier phase, $n_{l}=n_{l,c}+{\mathrm{jn}}_{l,s}$ additive Gaussian white noise (AWGN) with zero mean and variance $\sigma^{2}$.

The incoming samples are parsed into blocks of size 2N × 1. Two channels of incoming data samples in terms of vectors are $s_{0}={\left[s_{\tau +\mathrm{mN}+1},s_{\tau +\mathrm{mN}+2},\ldots ,s_{\tau +\mathrm{mN}+2N}\right]}^{T},$ $s_{1}={\left[s_{\tau +\mathrm{mN}+2N+1},s_{\tau +\mathrm{mN}+2N+2},\ldots ,s_{\tau +\mathrm{mN}+4N}\right]}^{T}$. Partitioning the samples into blocks with FN, folding the blocks F times to produce new blocks with length N and zero-padding each block to 2N gives $c_{\mathrm{xf},\sum }$ = $\left(\sum_{j=1}^{F}c_{\mathrm{jN}+1+\delta },\sum_{j=1}^{F}c_{\mathrm{jN}+2+\delta },\ldots ,\sum_{j=1}^{F}c_{\mathrm{jN}+N+\delta },0,\;0,\ldots ,\;0\right)$. Note that τ is the arbitrary initial code phase and δ is the offset.

In this paper, to simplify the models and simulations, it is assumed that no data toggle occurs during coherent accumulation, the circular correlation results is as follows:

(2) $$\begin{array}{l} R_{1,\delta }=\sum_{j=1}^{F}\sum_{l=1}^{2N}s_{l}c_{\mathrm{jN}+l+\delta }\\ =\sum_{j=1}^{F}\sum_{l=1}^{2N}\left({\mathrm{Ad}}_{l+\tau }c_{l+\tau }+n_{l}\right)e^{\left[\left(\frac{2{\pi f}_{d}}{f_{s}}\right)l+\varphi \right]}c_{\mathrm{jN}+l+\delta }\\ =\sum_{j=1}^{F}\sum_{l=1}^{2N}{\mathrm{Ad}}_{l}c_{l}e^{\left[\left(\frac{2{\pi f}_{d}}{f_{s}}\right)l+\varphi \right]}c_{\mathrm{jN}+l+\delta }+\sum_{j=1}^{F}\sum_{l=1}^{2N}n_{l}e^{\left[\left(\frac{2{\pi f}_{d}}{f_{s}}\right)l+\varphi \right]}c_{\mathrm{jN}+l+\delta } \end{array}$$

(3) $$\begin{array}{l} R_{2,\delta }=\sum_{j=1}^{F}\sum_{l=1}^{2N}s_{2N+l}c_{\mathrm{jN}+l+\delta }\\ =\sum_{j=1}^{F}\sum_{l=1}^{2N}\left({\mathrm{Ad}}_{2N+l+\tau }c_{2N+l+\tau }+n_{2N+l}\right)e^{\left[\left(\frac{2{\pi f}_{d}}{f_{s}}\right)l+\varphi \right]}c_{\mathrm{jN}+l+\delta }\\ =\sum_{j=1}^{F}\sum_{l=1}^{2N}{\mathrm{Ad}}_{2N+l+\tau }c_{2N+l+\tau }e^{\left[\left(\frac{2{\pi f}_{d}}{f_{s}}\right)l+\varphi \right]}c_{\mathrm{jN}+l+\delta }+\sum_{j=1}^{F}\sum_{l=1}^{2N}n_{2N+l}e^{\left[\left(\frac{2{\pi f}_{d}}{f_{s}}\right)l+\varphi \right]}c_{\mathrm{jN}+l+\delta } \end{array}$$

where F denotes folding number of local PN-codes and N the block length.

The correlation results is composed of two items: coherent integration and AWGN.

When the signal segment is indeed contained within the extended segment, the coherent term can be shown to be a Gaussian variable with a mean of N and variance of (F−1) × NA^2^, that is:

(4) $$\sum_{j=1}^{F}\sum_{l=1}^{2N}{\mathrm{Ad}}_{l}c_{l}e^{\left[\left(\frac{2{\pi f}_{d}}{f_{s}}\right)l+\varphi \right]}c_{\mathrm{jN}+l+\delta }\sim \psi \left(N,\left(F-1\right){\mathrm{NA}}^{2}\right)$$

When the signal segment is not contained within the extended segment, the distribution is as follows:

(5) $$\sum_{j=1}^{F}\sum_{l=1}^{2N}{\mathrm{Ad}}_{l}c_{l}e^{\left[\left(\frac{2{\pi f}_{d}}{f_{s}}\right)l+\varphi \right]}c_{\mathrm{jN}+l+\delta }\sim \psi \left(0,{\mathrm{FNA}}^{2}\right)$$

The probability distribution is obtained by the virtue of the central limit theorem under assumptions for large N.

The noise is treated as AWGN with zero mean and variance $\sigma^{2}$, so the summation of all the multiplication between noise and PN-code, that is, the AWGN term in correlation result, follows:

(6) $$\sum_{j=1}^{F}\sum_{l=1}^{2N}n_{2N+l+\tau }e^{\left[\left(\frac{2{\pi f}_{d}}{f_{s}}\right)l+\varphi \right]}c_{\mathrm{jN}+l+\delta }\sim \psi \left(0,{\mathrm{FN}\sigma }^{2}\right)$$

We can see from the formulas that the number of folds mainly effects the intensity of noise. It is the reason for the SNR deterioration of folding methods. Given the distribution of coherent integration and noise, we can subsequently infer the theoretical detection probability below.

### 3.2. Detection Probability

Instead of using threshold of the peak, the DC-XFAST method uses the maximum and second maximum value of correlation results and position of correlation peaks to judge if the acquisition process is succeeded. Provided that the carrier residual frequency is properly compensated, which means $f_{d}=0$, the correlation result is constituted by three items: coherent integration result, input-related noise and self-noise result from folding. Since real and imaginary parts of the mixing noise are uncorrelated Gaussian noise with zero mean and variance of $\sigma_{H}^{2}=\mathrm{NF}\left(A^{2}+\sigma^{2}\right)$, the absolute magnitude of the correlation result will be Rice distribution with probability density function of:

(7) $$P_{\mathrm{Ricean}}\left(r\right)=\frac{r}{{N\sigma }_{H}^{2}}\exp\left(-\frac{r^{2}+s_{k}^{2}}{2{N\sigma }_{H}^{2}}\right)I_{0}\left(\frac{{\mathrm{rs}}_{k}}{{N\sigma }_{H}^{2}}\right)$$

With $I_{0}$ the zero order modified Bessel function. $s_{k}=N$ is the summation of the mean of the signals on the in phase and quadrature branches for bin k. Partial correlation also follows the Rice distribution with $s_{k}$ = k for $R_{1,m-1,\delta }$, $s_{k}$ = N for $R_{1,m,\delta }$ and $s_{k}$ = N – k for $R_{1,m+1,\delta }$. After stacking the correlation result of m − 1 and m + 1, $s_{k}=N$, which means the magnitude of full peak which contained two partial peaks is not affected by k. The correlation result is shown in Figure 3.

When there are only self-noise and input-related noise in the coherent integration results, the absolute magnitude of the correlation result will be Rayleigh distribution with probability density function of:

(8) $$P_{\mathrm{Rayleigh}}\left(r\right)=\frac{r}{{N\sigma }_{H}^{2}}\exp\left(-\frac{r^{2}}{2{N\sigma }_{H}^{2}}\right),\;r\geq 0$$

And cumulative distribution function of:

(9) $$F_{\mathrm{Rayleigh}}\left(r\right)=1-\exp\left(-\frac{r^{2}}{2{N\sigma }_{H}^{2}}\right),\;r\geq 0$$

The probability of two peaks are detected simultaneously in a single cell is $P_{\mathrm{dc},2}\left(s_{k}\right)=\int_{0}^{\infty }\int_{0}^{\infty }P\left(n_{1}<r_{k1},r_{k2},\ldots n_{2N}<r_{k1},r_{k2}{|r}_{k1},r_{k2}\right)P_{\mathrm{Ricean}}\left(r_{k1}\right)P_{\mathrm{Ricean}}\left(r_{k2}\right){\mathrm{dr}}_{k1}{\mathrm{dr}}_{k2}$, $r_{k1}$ and $r_{k2}$ is signal items of the two peak in a single cell, $n_{i}$ represent noise items. Considering $r_{k}$ and $n_{i}$ follow Rice distribution and Rayleigh distribution respectively, the probability can be derived as follows:

(10) $$\begin{array}{l} P_{\mathrm{dc},2}\left(s_{k}\right)=\int_{0}^{\infty }\int_{0}^{\infty }P\left(n_{1}<r_{k1},r_{k2},\ldots n_{2N}<r_{k1},r_{k2}{|r}_{k1},r_{k2}\right)P_{\mathrm{Ricean}}\left(r_{k1}\right)P_{\mathrm{Ricean}}\left(r_{k2}\right){\mathrm{dr}}_{k1}{\mathrm{dr}}_{k2}\\ =2\int_{0}^{\infty }\int_{r_{k1}}^{\infty }P\left(n_{1}<r_{k1},\ldots n_{2N}<r_{k1}{|r}_{k1}\right)P_{\mathrm{Ricean}}\left(r_{k1}\right)P_{\mathrm{Ricean}}\left(r_{k2}\right){\mathrm{dr}}_{k1}{\mathrm{dr}}_{k2}\\ =2\int_{0}^{\infty }\int_{r_{k1}}^{\infty }\prod_{i=1,i\neq k1,k2}^{2N}P\left(n_{i}<r_{k1}{|r}_{k1}\right)P_{\mathrm{Ricean}}\left(r_{k1}\right)P_{\mathrm{Ricean}}\left(r_{k2}\right){\mathrm{dr}}_{k1}{\mathrm{dr}}_{k2}\\ =2\int_{0}^{\infty }\int_{r_{k1}}^{\infty }{\left[-\exp\left(-\frac{{r_{k1}}^{2}}{2{N\sigma }_{H}^{2}}\right)\right]}^{2N-2}\frac{r_{k1}}{{N\sigma }_{H}^{2}}\exp\left(-\frac{{r_{k1}}^{2}+s_{k}^{2}}{2{N\sigma }_{H}^{2}}\right)I_{0}\left(\frac{r_{k1}s_{k}}{{N\sigma }_{H}^{2}}\right)\frac{r_{k2}}{{N\sigma }_{H}^{2}}\exp\left(-\frac{{r_{k2}}^{2}+s_{k}^{2}}{2{N\sigma }_{H}^{2}}\right)I_{0}\left(\frac{r_{k2}s_{k}}{{N\sigma }_{H}^{2}}\right){\mathrm{dr}}_{k1}{\mathrm{dr}}_{k2} \end{array}$$

The probability of at least one peak is detected in a single cell can be derived as:

(11) $$\begin{array}{l} P_{\mathrm{dc},1}\left(s_{k}\right)=2\int_{0}^{\infty }\int_{0}^{r_{k1}}P\left(n_{1}<r_{k1},\ldots n_{2N}<r_{k1}{|r}_{k1}\right)P_{\mathrm{Ricean}}\left(r_{k1}\right)P_{\mathrm{Ricean}}\left(r_{k2}\right){\mathrm{dr}}_{k1}{\mathrm{dr}}_{k2}\\ =2\int_{0}^{\infty }\int_{0}^{r_{k1}}\prod_{i=1,i\neq k1,k2}^{2N}P\left(n_{i}<r_{k1}{|r}_{k1}\right)P_{\mathrm{Ricean}}\left(r_{k1}\right)P_{\mathrm{Ricean}}\left(r_{k2}\right){\mathrm{dr}}_{k1}{\mathrm{dr}}_{k2}\\ =2\int_{0}^{\infty }\int_{0}^{r_{k1}}{\left[-\exp\left(-\frac{{r_{k1}}^{2}}{2{N\sigma }_{H}^{2}}\right)\right]}^{2N-2}\frac{r_{k1}}{{N\sigma }_{H}^{2}}\exp\left(-\frac{{r_{k1}}^{2}+s_{k}^{2}}{2{N\sigma }_{H}^{2}}\right)I_{0}\left(\frac{r_{k1}s_{k}}{{N\sigma }_{H}^{2}}\right)\frac{r_{k2}}{{N\sigma }_{H}^{2}}\exp\left(-\frac{{r_{k2}}^{2}+s_{k}^{2}}{2{N\sigma }_{H}^{2}}\right)I_{0}\left(\frac{r_{k2}s_{k}}{{N\sigma }_{H}^{2}}\right){\mathrm{dr}}_{k1}{\mathrm{dr}}_{k2} \end{array}$$

For clarity, the probabilities can be written as $P_{2}=P_{\mathrm{dc},2}\left(N\right)$ and $P_{1}=P_{\mathrm{dc},1}\left(N\right)$. According to the judging strategy of DC-XFAST, the result will be positive if the number of peaks in certain positions is more than $N_{\mathrm{th}}$ which can be chosen from 1 to 4. The detection probabilities with different threshold is as follows.

$N_{\mathrm{th}}=4$, since the correlation results of each cell is independent, the probability of all correct detection is the multiplication of the probability of two peaks being detected simultaneously in a single cell:

(12) $$P_{\mathrm{dc}}{|}_{N_{\mathrm{th}}=4}=P_{2}^{2}$$

$N_{\mathrm{th}}=3$, the probability of equal to or more than three peaks being correctly detected is:

(13) $$P_{\mathrm{dc}}{|}_{N_{\mathrm{th}}=3}=2P_{2}\left(P_{1}-P_{2}\right)+P_{2}^{2}$$

$N_{\mathrm{th}}=2$, the probability of equal to or more than three peaks being correctly detected is:

(14) $$P_{\mathrm{dc}}{|}_{N_{\mathrm{th}}=2}=2P_{2}\left(1-P_{1}\right)+{\left(P_{1}-P_{2}\right)}^{2}+2P_{2}\left(P_{1}-P_{2}\right)+P_{2}^{2}$$

$N_{\mathrm{th}}=1$, the probability that all correct peaks miss detection is ${\left(1-P_{1}\right)}^{2}$, then the probability of at least one peak being detected is as follows:

(15) $$P_{\mathrm{dc}}{|}_{N_{\mathrm{th}}=1}=1-{\left(1-P_{1}\right)}^{2}$$

A smaller threshold $N_{\mathrm{th}}$ results in a larger number of combinations, thus a higher chance of better detection performance is ensured.

### 3.3. Probability of False Alarm

A false alarm arises when the number of noise peaks with a specific relative position is greater than or equal to $N_{\mathrm{th}}$. The greater the threshold, the lower the false alarm probability, for example, if the threshold is chosen as 4, which means four peaks with relative position have been detected. Since noises within each cell are independent, the probability that the noise value at any certain position is the largest value within a cell is 1/N, and the total number of occurrences amounts to $N^{4}$. Denoting the false alarm probability based on max-value detection as $P_{\mathrm{fa}}$, one can write the expressions of false alarm probability in each $N_{\mathrm{th}}$ as $N_{\mathrm{th}}=4$, so when four noise peaks with relative position of 0 or N are detected, then a false alarm arises. Since the probability that the noise value at any certain position is the largest value within a cell is 1/N, the probability that four noise values arise simultaneously is 1/$N^{4}$. Considering the total number of possible positions is N, the false alarm probability is derived as:

(16) $$P_{\mathrm{fa}}{|}_{N_{\mathrm{th}}=4}=P_{\mathrm{fa}}\frac{N}{N^{4}}=P_{\mathrm{fa}}\frac{1}{N^{3}}$$

$N_{\mathrm{th}}=3$, the probability that three noise peaks with detected relative position is:

(17) $$P_{\mathrm{fa}}{|}_{N_{\mathrm{th}}=3}=P_{\mathrm{fa}}\frac{4N\left(N-1\right)+N}{N^{4}}$$

$N_{\mathrm{th}}=2$, the probability that two noise peaks with relative detected position is:

(18) $$P_{\mathrm{fa}}{|}_{N_{\mathrm{th}}=2}=P_{\mathrm{fa}}\frac{4N\left(N-1\right)+6N{\left(N-1\right)}^{2}+N}{N^{4}}$$

$N_{\mathrm{th}}=1$, the probability that one noise peaks with detected relative position is:

(19) $$P_{\mathrm{fa}}{|}_{N_{\mathrm{th}}=1}=P_{\mathrm{fa}}$$

### 3.4. Mean Acquisition Time

Since the analysis models for mean acquisition time of zero-padding, XFAST, DF-XFAST and DC-XFAST are similar and all based on $P_{d}$ and $P_{\mathrm{fa}}$, a common formula could be given. Only single dwell time is considered and FFT operation is assumed to be used in code phase search for acquisition that means the process time for each correlation is equal to chip rate. The mean acquisition time normalized to system clock can be written as follows:

(20) $$\bar{T_{a}}=\frac{1}{P_{d}}+\frac{2-P_{d}}{2P_{d}}\left(\lfloor \frac{\mathrm{LM}}{\mathrm{qN}}\rfloor -1\right)\left(1+{\mathrm{qkP}}_{\mathrm{fa}}\right)$$

q denotes the number of blocks that can be handled in each process. For XFAST, DF-XFAST and DC-XFAST, q usually takes number of local code segment moved by one step, usually takes local code segment folding times. For the zero-padding method, q = 1. L denotes a long PN-code period, N the block length, M the samples on each chip and k the penalty time index. The penalty time index for folding methods like DF-XFAST, XFAST and DC-XFAST are q times the zero-padding method one due to detection ambiguity.

## 4. Simulation Results

### 4.1. Simulation Parameters

In this part, we present the numerical results of coarse acquisition for the detection probabilities, mean acquisition time and acquisition performance comparisons during zero-padding, XFAST, DF-XFAST and DC-XFAST, as a function of SNR for fixed false alarm probabilities. Both Monte Carlo results and theoretical results are given when discussing the detection probabilities.

Though inter-satellite link ranging mode in both GPS and BDS adopt a long PN-code spread spectrum system. GPS IIR satellite ranging mode uses the ultrahigh frequency (UHF) band to measure distance, it applies a broadcast mode to communicate with other satellites [29], while BDS chooses the Ka band, which is an end-to-end way for satellite communication. The transmission of the former can provide additional observation information. Thus the PN-code of GPS ranging mode of inter-satellite link is employed to check the acquisition performance, the PN-code period of which is $2^{17}-1$ with chip rate 5 MHz, and the generator polynomial is G(X)=$1+X^{3}+X^{17}$. Since the SNR range of GPS inter-satellite link is −21 dB~−1 dB [30], SNR settings for simulation range from −25 dB~0 dB. To simplify the computation, sampling frequency $f_{s}$ is set as 5 MHz and residual code phase offset is set as zero. The quantity of chip for FFT($N_{\mathrm{chip}}$) is 4096.

The false alarm probability $P_{\mathrm{fa}}$ is determined by the choice of threshold, assumed that single peak false alarm probability is $10^{-5}$, the false alarm probabilities of each threshold $N_{\mathrm{th}}$ is presented in the following Table 1.

### 4.2. Detection Performance

**Figure 4:**
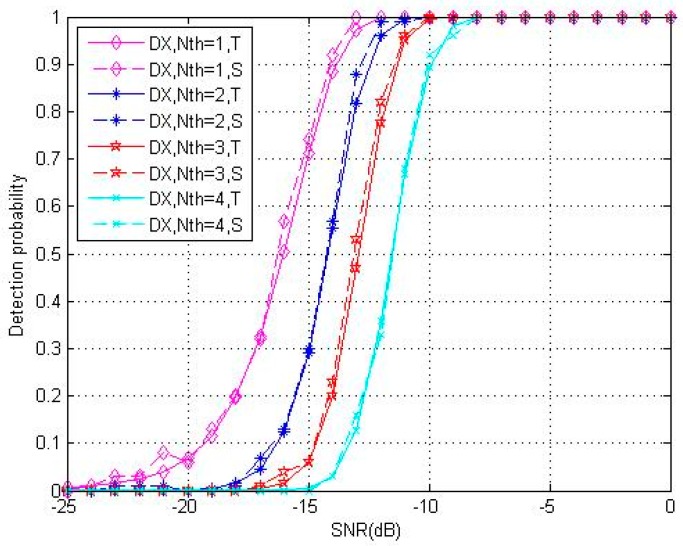
Theoretical (“T”, continuous line) and simulation (“S”, dotted line) results for detection probability under 1–4 verification times ($N_{th}$) of DC-XFAST (“DX”) with folding number equals to 10.

This paper discussed in detail the relationship between $P_{d}$ and $N_{\mathrm{th}}$ in the third part. Figure 4 displays a group of theoretical and simulation detection probabilities with different $N_{\mathrm{th}}$(N = 4096, F = 10). The curve moves to right about 1.3 dB for every $N_{\mathrm{th}}$ increase, and the approach with $N_{\mathrm{th}}=1$ has more advantage in $P_{d}$. The greater the threshold, the lower the false alarm probability and detection probability. Therefore, applications with different requirements should consider the balance of $P_{d}$ and $P_{\mathrm{fa}}$. In addition, Figure 4 shows that even though the detection probability declines when $N_{\mathrm{th}}$ increases, DC-XFAST does not deteriorate badly when the number of verification times reaches 4. The reason is that the optimal structure design assures proper use of partial peaks, and the magnitude of peaks which contain two partial peaks is the same as that of a full peak.

The detection performance of folding methods like XFAST, DF-XFAST and DC-XFAST is degraded from folding. The proposed method uses a max-value tactic to ensure higher detection probability than XFAST and DF-XFAST, which are based on a constant false alarm probability. Besides, DC-XFAST considers partial peaks, so multi-peak verification can effectively avoid peak ambiguity.

**Figure 5:**
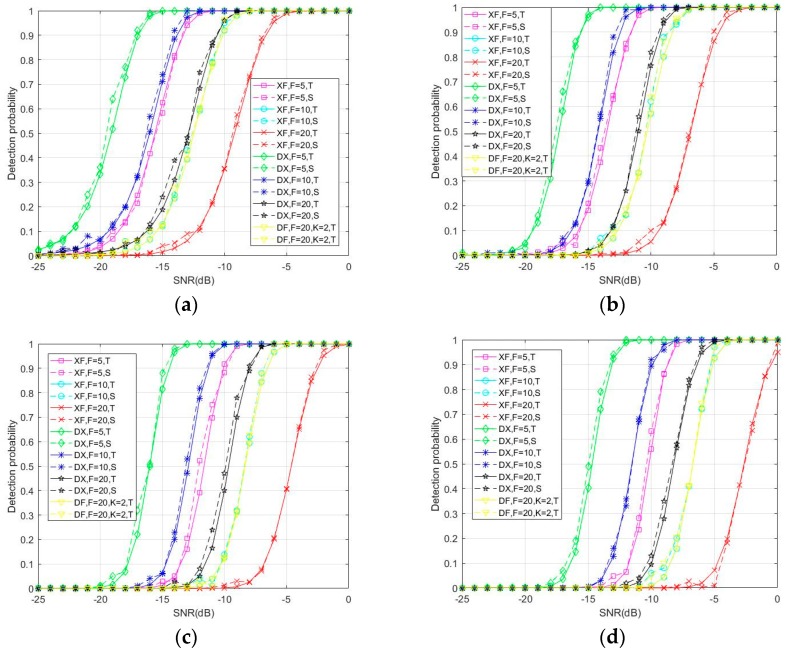
Theoretical (“T”, continuous line) and simulation (“S”, dotted line) results for detection probability under 1–4 verification times ($N_{\mathrm{th}}$) of DC-XFAST (“DX”), DF-XFAST (“DF”) and XFAST (“XF”) with folding number equals to 5, 10 and 20 and $N_{\mathrm{th}}$ equals to 1(**a**), 2(**b**), 3(**c**), 4(**d**).

Figure 5 reports the advantage of the DC-XFAST method over XFAST and DF-XFAST with regard to detection probability when the number of verification times is from 1~4. The figure demonstrates that, compared with XFAST, with the same local folding number, DC-XFAST has improved detection performance and the gain is about 4 dB. The greater the folding number, the more obvious the comparative advantage DC-XFAST can achieve, and when the folding number is 20, the gain is 6 dB and 7 dB, respectively, when $N_{\mathrm{th}}$ = 3 and 4. Even when the folding number of XFAST is five times more than that of DC-XFAST, the acquisition performance improvements of DC-XFAST are noticeable. DC-XFAST outperforms DF-XFAST in detection performance as well. Especially when $N_{\mathrm{th}}=4$ and F = 20, the gain is about 2 dB. To conclude, as the folding number and number of verification times increase, DC-XFAST shows greater advantage over the other folding methods XFAST and DF-XFAST.

### 4.3. Acquisition Performance

Since it is hard to give an analysis in a meantime-SNR graph, only theoretical results were given in this paper. Moreover, we assumed that the unit of mean acquisition time is the time taken by one correlation calculation.

**Figure 6:**
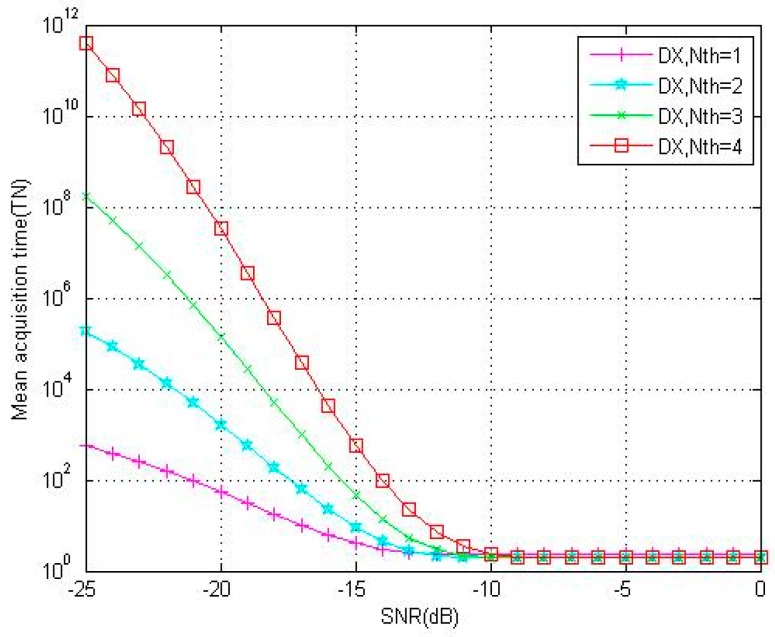
Theoretical results for mean acquisition time under 1–4 verification times ($N_{\mathrm{th}}$) of DC-XFAST (“DX”).

**Figure 7:**
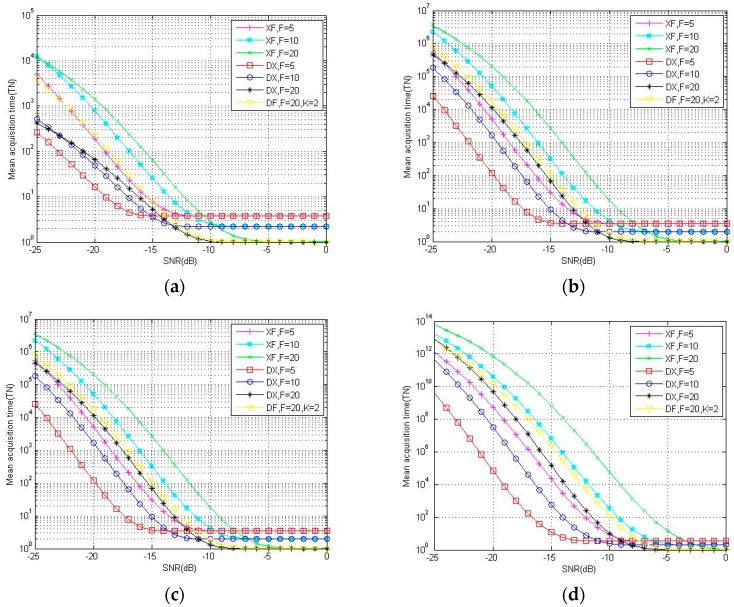
Theoretical results for mean acquisition time of DC-XFAST (“DX”), DF-XFAST (“DF”) and XFAST (“XF”) with N equals to 4096 and $N_{\mathrm{th}}$ equals to 1(**a**), 2(**b**), 3(**c**), 4(**d**).

Figure 6 plots the theoretical mean acquisition time of DC-XFAST with different verification times ($N_{\mathrm{th}}$). As discussed in the third part, analysis models for mean acquisition time of these methods are all based on $P_{d}$ and $P_{\mathrm{fa}}$, and the greater the threshold, the lower the false alarm probability and detection probability. The figure shows that even when the false alarm probability is lower, the low detection probability of models with higher verification times could not be counteracted, thus the mean acquisition time is longer. Thus, the application of DC-XFAST should balance false alarm and time cost. Figure 7 illustrates the acquisition performance of three folding methods as mean acquisition time. The four figures all show that when the incoming signal is weak, that is, the incoming SNR is between −25 dB and −10 dB, folding degrades the PN-code correlation properties and the degradation is reflected by the mean acquisition time as well. By the position verification of multi-peaks, DC-XFAST decreases the false alarm probability and thus diminishes the degradation of folded PN-code correlation properties. With the same local folding number, and when the SNR is not larger than −15 dB, the mean acquisition time of DC-XFAST is about 1/10 that of XFAST, and about 1/5 that of DF-XFAST when $N_{\mathrm{th}}$ is 3 and 4. When the incoming SNR is larger than −10 dB, with a larger folding number, the code phases searched in parallel are increased and the benefit of folding is represented in the decrease of mean acquisition time.

**Figure 8:**
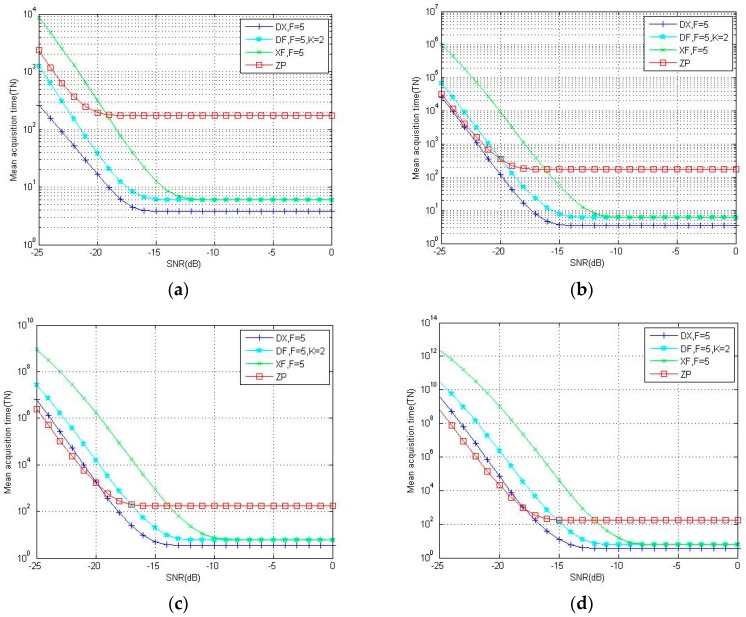
Theoretical results for mean acquisition time of Zero-padding (“ZP”), DC-XFAST (“DX”), DF-XFAST (“DF”) and XFAST (“XF”) with N equals to 4096, F equals to 5 and $N_{th}$ equals to 1(**a**), 2(**b**), 3(**c**), 4(**d**).

Figure 8 focuses on the acquisition performance analysis between the folding method and unfolding method. By folding the local signal, folding methods like XFAST, DF-XFAST and DC-XFAST decrease the cardinality of the code phase, hence, when the SNR is higher than −15 dB, folding methods consume less acquisition time than ZP. However, when the SNR is lower than −18 dB, the benefit of folding decreases and the cost appears. As shown in Figure 8, XFAST and DF-XFAST with weak signal have longer mean acquisition time than ZP. With respect to the proposed DC-XFAST method, DC-XFAST shows a noticeable advantage over XFAST and DF-XFAST in a low SNR environment. Especially when $N_{\mathrm{th}}$ equals to 1 and 2, the mean acquisition time of DC-XFAST is about 1/50 that of ZP. When $N_{\mathrm{th}}=$ 3 and 4, the advantage of multi-peak verification cannot compensate for the degradation of folding, DC-XFAST shows a slight disadvantage compared with ZP when the SNR is less than −20 dB, but as shown in Figure 8c,d, when the SNR is between −20 dB and 0 dB, DC-XFAST consumes much less acquisition time than ZP.

## 5. Discussion

Inter-satellite links are characterized by low signal-to-noise ratio (SNR), complex electromagnetic interference, and the time slot of each satellite being only 1.5 s. These all factors bring great difficulties to long code acquisition in inter-satellite links.

In addition, with the rapid development of inter-satellite links, the number of satellites is on the increase and as a result, the electromagnetic environment is becoming more and more complex [31]. It is to be expected that the period PN-code would increase in the face of increasingly complex electromagnetic environment. Thus, studying long PN-code acquisition in inter-satellite links has a significant meaning to the theory and practices of inter-satellite link development.

The proposed method uses multi-peak verification to guarantee lower false alarm probability, and thereby, to some extent, avoid SNR deterioration result from folding and provide a more stable detection performance than folding methods like XFAST and DF-XFAST.

### 5.1. System Model and Complexity Burden

The Max-value tactic is applied for detection. The verification part of DC-XFAST is less complex than an architecture that combines over-threshold and max-value detection. The proposed verification tactic does not have any magnitude or power detection, and only the relative position of each peak is of interest. Thus, eliminating the signal power and/or noise power estimations is indispensable in fixed or unfixed threshold approaches.

In addition, with proper timing control, two channels can be processed sequentially instead of in parallel. As reflected by the system model in Figure 1, two channels share the same correlator and do not put any extra burden on the computation complexity and the resulting brief delay in the inter-satellite links is acceptable.

Three FFT modules are instantiated in total. The incoming signal blocks in two channels and local block would perform a 2N points FFT. Their multiplication would perform a 2N points IFFT. The procedure includes five FFTs and the FFT operations are implemented with $5{\mathrm{Nlog}}_{2}2N$ complex multiplications and $10{\mathrm{Nlog}}_{2}2N$ complex additions in total. The XFAST procedure includes three FFTs and the FFT operations are implemented with $3{\mathrm{Nlog}}_{2}2N$ complex multiplications and $6{\mathrm{Nlog}}_{2}2N$ complex additions in total. However, the search range of DC-XFAST is twice the size of that of XFAST, thus DC-XFAST does not increase the computational complexity.

### 5.2. Parameter Analysis

Applications with different requirements should consider the balance of $P_{d}$ and $P_{\mathrm{fa}}$. The greater the threshold, the lower the false alarm probability and detection probability. If the system requires a lower false alarm rate, a higher verification time is suggested, and if the system requires faster acquisition, a lower verification time is recommended. For general applications, $N_{\mathrm{th}}=2$ is recommended, whereby the false alarm probability would be under $10^{-5}$, which is low enough for most cases.

### 5.3. Method Comparison

By integrating the dual-channel method and XFAST method, we have developed a new method that has the advantages of both, although the integration is not a simple combination.

Like the dual-channel method, the byproduct of correlation, partial peaks, are considered in the acquisition verification instead of being neglected. The difference being, in DC-XFAST, that partial peaks are stacked so that the detection performance is not affected by their magnitude. Moreover, the entire local code segment is folded to have half of the incoming signal block length, and folding local replicas are zero-padded to full length. The use of the folding technique reduces the acquisition time.

Both the DC-XFAST method and XFAST method adopt folding techniques to achieve rapid acquisition, but the DC-XFAST method applies max-value detection to achieve a high detection probability. Moreover, DC-XFAST performs the subsequent multi-peak verification, which is the core step of DC-XFAST to address SNR deterioration resulting from folding.

## 6. Conclusions

This paper has presented a dual-channel acquisition method that is based on the extended replica folding algorithm (DC-XFAST) method for long PN-code acquisition. To make up for the SNR deterioration resulting from folding and false alarm dues to peak position ambiguity, the proposed method uses multi-peak verification to guarantee a low false alarm probability and applies max-value detection to achieve a high detection probability. Compared with the unfolding method ZP, DC-XFAST takes advantage of reducing the code phase to be searched by folding the local PN-code to shorten the mean acquisition time. Compared with the folding methods XFAST and DF-XFAST, DC-XFAST has a stable detection probability and a lower false alarm probability. Benefiting from its multi-peak secondary verification, DC-XFAST requires much less mean acquisition time than other methods. The detailed structure of this method is described and its performance is analyzed.

The enhancement of DC-XFAST with respect to DF-XFAST, XFAST and ZP has been demonstrated by numerical results. The best acquisition probability gain of DC-XFAST method would be about 7 dB compared with XFAST and 2 dB compared with DF-XFAST when the folding number is 20 and the threshold is 4. In addition, with the advantage of higher detection probability and lower false alarm probability, the mean acquisition time of DC-XFAST would be about 1/10 that of XFAST, 1/5 that of DF-XFAST and 1/50 that of zero-padding.

Numerical results show that the performance of DC-XFAST is optimized as a whole. In the situation of complex electromagnetic interference and low SNR, this method can achieve a stable detection probability and a low false alarm probability. Moreover, this method takes much less acquisition time than the conventional methods when the time-slot is short, therefore, DC-XFAST is a viable solution for long code acquisition in inter-satellite links.

## Figures and Tables

**Table 1:** The false alarm probability $P_{\mathrm{fa}}$ of each threshold $N_{\mathrm{th}}$.

$N_{\mathrm{th}}$	1	2	3	4
$P_{\mathrm{fa}}$	$10^{-5}$	1.46 × $10^{-8}$	2.38 × $10^{-12}$	1.46 × $10^{-16}$
